# The Reach, Use, and Impact of a Free mHealth Mindfulness App in the General Population: Mobile Data Analysis

**DOI:** 10.2196/23377

**Published:** 2020-11-27

**Authors:** Elissa Kozlov, Erin Bantum, Ian Pagano, Robyn Walser, Kelly Ramsey, Katherine Taylor, Beth Jaworski, Jason Owen

**Affiliations:** 1 Institute for Health, Health Policy and Aging Research Rutgers University New Brunswick, NJ United States; 2 School of Public Health Rutgers University New Brunswick, NJ United States; 3 Cancer Prevention in the Pacific University of Hawaii Cancer Center Honolulu, HI United States; 4 National Center for PTSD Research VA Palo Alto Healthcare System Menlo Park, CA United States

**Keywords:** mHealth, mindfulness, mHealth psychotherapy, mHealth mindfulness, public health, self-management, mental health

## Abstract

**Background:**

As smartphones are now used by most Americans, it is increasingly possible for mental health mobile apps to be disseminated to the general public. However, little is known about how mobile mental health apps are used by the general population outside of a controlled research design.

**Objective:**

Our objective is to describe how the general population engages with Mindfulness Coach, an iOS- and Android-based app designed to deliver a mindfulness training course.

**Methods:**

Using anonymous download and analytics data, we characterized the reach, usage, retention, and impact of Mindfulness Coach. We included mobile analytics data from all unique downloads of Mindfulness Coach between August 1, 2018, and April 8, 2019 (N=104,067) as well as starred reviews from all Mindfulness Coach users who provided reviews of the app as of March 1, 2020. Mindfulness characteristics were measured by an in-app assessment using the Five-Facet Mindfulness Questionnaire–Short Form (FFMQ-SF).

**Results:**

Users engaged, on average, in 4.3 visits to the app (SD 8.8; median 2; 90th percentile 8) and associated with an average total of 49.2 interactions with the app (ie, clicks within the app) (SD 113.8; median 19; 90th percentile 105). Users spent an average of 16.2 minutes (SD 63.1) engaged with the app over the full study time period. There were strong linear effects of app engagement on total FFMQ-SF scores. For example, FFMQ-SF scores were associated with more time spent engaged with the app (*R*^2^=.23; *P*<.001). Mindfulness Coach has been reviewed in the Google Play Store 3415 times, with an average rating of 4.7 out of 5 stars, and over 2000 times in the Apple App Store, with an average rating of 4.8 out of 5 stars.

**Conclusions:**

These findings suggest that Mindfulness Coach has achieved substantial and sustained reach in the general population; however, it was used less frequently by many downloaders than researchers and designers intended. There was a subpopulation of users who engaged in the app regularly over an extended period of time, and there was a clear relationship between app use and improvements in mindfulness. To strengthen Mindfulness Coach’s public health impact, more research is needed to understand who is using the app and how, and to design strategies to increase user engagement in order for users to receive a larger dose of mindfulness treatment.

## Introduction

A recent National Academy of Medicine report recommended increased emphasis on disseminating and implementing evidence-based psychotherapies [1] in order to have maximal public health impact. Mindfulness therapy (MT) is a promising, nonpharmacological approach to manage various types of psychological distress [2-5]. Using guided meditation, psychoeducation, and targeted exercises, MT teaches people how to pay attention to the present moment without judgment. MT has proven efficacy and effectiveness in reducing anxiety and posttraumatic stress symptoms in diverse populations [2-4,6,7]. While the evidence base for MT is growing, traditional MT (ie, 8 or more sessions of face-to-face treatment with trained providers [2]) is likely not a realistic treatment model for the general population due to the lack of trained personnel, time constraints, reimbursement issues, and patient and provider availability [8,9]. An innovative delivery model is required to overcome these barriers. Mobile health (mHealth) can be useful to deliver behavioral interventions, as it surmounts many obstacles to traditional psychotherapy [10-12].

There is growing evidence that mHealth apps are an effective mechanism to deliver accessible mental health care [11]. Various studies demonstrate that mHealth interventions are both feasible and effective in teaching adults with depression or anxiety skills to manage their symptoms [13-18] and can be helpful to teach skills to manage other conditions, such as chronic pain [19-22]. There is preliminary evidence that apps can be utilized to disseminate aspects of MT so that individuals can learn and practice aspects of mindfulness on their own [23-25]; however, little is known about how mHealth-delivered MT is used by the general population without individualized guidance from practitioners or outside of a controlled research design.

It is essential that we better understand how mHealth strategies to manage psychological distress can be beneficial to the general population. Mobile mental health apps have the potential to reach millions of individuals who are unable to access individualized face-to-face or video therapy services [19,20]. There are many mobile mental health apps currently available, but while research is emerging on how apps work in optimal, highly supported conditions [12,21,22,26,27], less is known about how apps are used by the general population without additional input from providers. For example, what is the most efficacious dosage and duration of use, and how are people inclined to use the app without specific guidance? Without information about general public use patterns, it will be difficult to design apps to reach specific populations and address specific conditions and needs. In order to optimize and tailor user experiences with an app, we must understand how the app is used without instruction or guidance. This information has the potential to inform developers and clinicians about how users naturally engage with apps, offering the opportunity to develop targeted recommendations for enhanced use. Prior research with mental health mobile apps has revealed that many users will download the app and use it only once [28]. Further research on natural use and attrition patterns will allow developers to develop strategies to enhance sustained usage, such as setting in-app reminders to encourage sustained usage.

The purpose of this paper is to describe how the general population currently engages with Mindfulness Coach, an iOS- and Android-based app designed to deliver a mindfulness training course based on the adaptation of several US Department of Veterans Affairs (VA) protocols. Based on anonymous download and analytics data, we aim to characterize the reach, usage, retention, and impact of Mindfulness Coach, a publicly available mobile app.

## Methods

### Overview

Mindfulness Coach is a native iOS- and Android-based app designed to deliver mindfulness training adapted from several VA protocols. The app is intended to provide a highly engaging introduction to MT and is tailored to users who may be skeptical about meditation practices by providing simple instructions and brief exercises. After downloading the app, users are provided with a brief tutorial that introduces the major features (ie, training plan, practice exercises, learning topics, and tracking). The training plan attempts to provide users with direction on how to use the app by gently introducing the user to each of the components within the app. Users can set push notification reminders in the settings section if they choose. The app delivers 14 sessions (ie, levels), each culminating in a meditative exercise. The app provides a training plan, evidence-based mindfulness audio exercises, assessment using the Five-Facet Mindfulness Questionnaire (FFMQ) [29], and education about MT. The app transmits deidentified usage data to a secure server using methods approved under the VA’s Technical Reference Model [30].

### Data Sources

Data were derived from 2 sources. First, we included mobile analytics data from all unique downloads of Mindfulness Coach between August 1, 2018, and April 8, 2019 (N=104,067). Second, we included starred reviews from all Mindfulness Coach users who provided reviews of the app on either the Apple App Store or the Google Play Store as of March 1, 2020.

### Measures

#### The Five-Facet Mindfulness Questionnaire–Short Form

The FFMQ is a measure of the 5 facets of the tendency to be mindful in daily life: observing, describing, acting with awareness, nonreactivity to inner experience, and nonjudging of inner experience. As a measure of impact, the app collects data on the FFMQ-Short Form (FFMQ-SF). The app’s training plan recommends that users complete the FFMQ-SF at levels 1, 7, and 14, and they are provided prompts to do so. Additionally, participants can take the assessment whenever they want by clicking the *Track my Progress* button. While taking the assessment is recommended, it is not required, as users can close out of the assessment at any time. Mindfulness Coach administers only 4 of the FFMQ-SF’s 5 subscales: *being observant*, *acting with awareness*, *nonjudging*, and *nonreactivity*. The *describing* subscale was not used because Mindfulness Coach does not provide tools for improving communication skills. The FFMQ-SF without the *describing* subscale consists of 19 items, each measured on a 5-point Likert scale, ranging from 1 (*Never or very rarely true*) to 5 (*Very often or always true*). Each of the 4 subscales has been shown to have adequate internal consistency (α values from .75 to .83) and comparability to the full 39-item FFMQ scale, with strong divergent and convergent validity and sensitivity to change over time [31,32].

#### Mobile Analytics

For each platform, iOS or Android, we captured basic user engagement measures (ie, number of downloads, active users, number of events within the app during each visit, visit duration, and number of visits) in addition to 2 primary measures of retention across time. Upon initial use of Mindfulness Coach, the app generates a unique, randomly generated string for that installation that is then associated with user engagement measures. The app did not collect any identifiers, such as IP address, location, device identifiers, or phone numbers, nor any other personal information (eg, battery state, data connections, etc) that could be used to identify an individual user [33].

Return use was calculated as the proportion of users who returned to use the app within 1 week of initial download, 1 month of initial download, 3 months of initial download, 6 months of initial download, and 12 months of initial download. Rolling retention was measured as the number of active days, weeks, and months of use of the app between the time of initial download and final use of the app during the observation period. Finally, sequences of events undertaken within the app were used to capture users’ navigation through the content pages of the app across time. Fully nonidentifying, anonymous, and encrypted event sequences were stored using JavaScript Object Notation (JSON) format on a remote Amazon Web Services GovCloud server. The event sequence data contained 9,170,219 records and were parsed using Perl regular expressions in SAS 9.4 (SAS Institute Inc) software. First-time users (ie, those who accepted the end-user license agreement) and returning users were identified. Each session began with the launch of the app and was classified as either a first-time use or a return visit on the basis of whether the end-user license agreement was displayed at launch or not.

For each session type (ie, first-time use vs return visit), specific usage events were tracked, including completing the app orientation, navigation from the home screen to 1 of the 4 primary content areas (ie, training plan, practice now, building expertise, or track progress), and navigation from one content area to another. We capped events at 30 minutes, unless the user had specified a longer value. We defined *visits* as clusters of events less than 30 minutes apart. Each record contained a field for its duration, and we defined *events* as records with nonzero values in the field, indicating that the user spent some amount of time with the app.

Means were determined by first calculating the within-subject means so that each subject’s average contributed equally to the grand mean. We considered the alternate method of calculating each visit’s sessions, but then users with more visits would contribute more to the grand mean, which could obfuscate potentially relevant data.

### Analyses

SAS 9.4 (SAS Institute Inc) software was used to perform all data management and analyses. We calculated descriptive statistics for usage data and the content areas visited. The MIXED procedure ran polynomial (ie, linear, quadratic, and cubic) repeated-measures regression analyses on the FFMQ-SF outcome. The predictor variables were the totals for retention time (ie, time spent in the app), number of visits, number of events, and highest mindfulness level achieved. We ran a separate model for each predictor given a high degree of multicollinearity among the predictor variables.

To further interpret and simplify the data, we created 4 categories of users: those who opened the app only once (*exploratory users*), those who visited the app 2 to 3 times (*limited users*), those who visited the app 4 to 7 times (*moderate* *users*), and those who visited the app 8 or more times (*committed users*). Categories were identified based on expected clinical benefit and also provided cut points that provided 4 roughly equal groups in terms of numbers of users in each group.

## Results

### Reach of Mindfulness Coach

We analyzed 104,067 unique downloads: 62.90% (65,458/104,067) on Android devices and 37.10% (38,609/104,067) on iOS devices. The app has been downloaded by an average of 6720 users per month since its release to the public on January 2019, with the number of users increasing steadily over time (ie, average of 9737 each month since December 2019). The total number of downloads was 278,606 since the app’s release on iOS in January 2014 and 147,535 since its release on Android in February 2018. Data available from the Apple App Store for iOS devices suggest that 94% of users accessed the app from a phone and 6% accessed the app from a tablet device (eg, an iPad).

### Satisfaction

Mindfulness Coach has been reviewed in the Google Play Store 3415 times, with an average rating of 4.7 out of 5 stars, and over 2000 times in the Apple App Store, with an average rating of 4.8 out of 5 stars.

### Use of the App

Elapsed time between first and final uses of the Mindfulness Coach app averaged 4.1 weeks (SD 6.7; median 0; 90th percentile 15). Users engaged, on average, in 4.3 visits to the app (SD 8.8; median 2; 90th percentile 8) and associated with an average total of 49.2 interactions with the app (ie, clicks within the app) (SD 113.8; median 19; 90th percentile 105). Users spent an average of 16.2 minutes (SD 63.1) engaged with the app over the full study time period. See Table 1 for the breakdown of use by user category and Table 2 for distribution of use by user category.

**Table 1:** Descriptive statistics of use variables by user visits to the app.

Visits, n	Downloads, n	License accepted, n (%)	Level achieved, mean (SD), range	Retention weeks, mean (SD), range	Active minutes, mean (SD), range	Visits, mean (SD), range	Average visit, mean (SD), range	Events, mean (SD), range
All	104,067	Total: 91,371 (87.80) Android: 66,499 (63.90) iOS: 38,609 (37.10)	1.4 (1.4), 1-14	4.1 (6.7), 0-34	16.2 (63.1), 9-5006	4.3 (9.5), 1-506	3.2 (4.4), 0-155	49.2 (113.8), 1-7597
1	40,544	Total: 34,908 (86.10) Android: 25,543 (63.00) iOS: 15,001 (37.00)	1.1 (0.3), 1-10	0 (0), N/A^a^	3.2 (5.3), 0-155	1 (0), N/A	3.2 (5.3), 0-155	15.4 (18.1), 1-275
2-3	32,962	Total: 29,402 (89.20) Android: 20,832 (63.20) iOS: 12,130 (36.80)	1.1 (0.5), 1-14	3.8 (5.6), 0-31	7.3 (9.4), 0-203	2.4 (0.5), 2-3	3.1 (3.9), 0-101	29.8 (28.9), 1-617
4-7	18,528	Total: 16,620 (89.70) Android: 11,543 (62.30) iOS: 6985 (37.70)	1.4 (0.9), 1-14	7.8 (7.3), 0-32	16.3 (17.7), 0-340	5.1 (1.1), 4-7	3.2 (3.4), 0-82	57.1 (48.8), 1-2003
≥8	12,033	Total: 10,421 (86.60) Android: 7557 (62.80) iOS: 4476 (37.20)	2.9 (3.3), 1-14	12.8 (8.4), 0-34	84.8 (167.6), 0-5006	19.3 (22.3), 8-506	4 (3.6), 0-46	204.1 (275.7), 1-7597

^a^N/A: not applicable; all values of the data set are the same, hence, there is no range.

**Table 2:** Distribution of use variables by user groups.

Use variables		Downloads, n	25th percentile	50th percentile	75th percentile	90th percentile
**Level achieved**						
	All	104,067	1	1	1	2
	1 visit	40,544	1	1	1	1
	2-3 visits	32,962	1	1	1	2
	4-7 visits	18,528	1	1	1	2
	≥8 visits	12,033	1	1	3	7
**Retention weeks**						
	All	104,067	0	0	5	15
	1 visit	40,544	0	0	0	0
	2-3 visits	32,962	0	1	5	12
	4-7 visits	18,528	2	5	12	19
	≥8 visits	12,033	5	12	20	25
**Active minutes**						
	All	104,067	1	4	13	32
	1 visit	40,544	0	1	4	9
	2-3 visits	32,962	1	4	10	18
	4-7 visits	18,528	4	11	22	37
	≥8 visits	12,033	18	40	89	184
**Visits**						
	All	104,067	1	2	4	8
	1 visit	40,544	1	1	1	1
	2-3 visits	32,962	2	2	3	3
	4-7 visits	18,528	4	5	6	7
	≥8 visits	12,033	9	12	20	36
**Events**						
	All	104,067	7	19	53	105
	1 visit	40,544	4	8	18	42
	2-3 visits	32,962	9	19	45	66
	4-7 visits	18,528	22	48	79	115
	≥8 visits	12,033	69	130	240	440

### Retention of Users

We used mobile analytics to characterize average use patterns as well as patterns of use over time. First, we investigated the proportion of users who actively used the app over time. Among individuals who downloaded and opened Mindfulness Coach between August 1, 2018, and April 8, 2019, 54.20% (56,404/104,067) used the app at least once after the first day it was installed, 43.20% (44,957/104,067) used the app at least once beyond the first week when it was downloaded, 30.40% (31,636/104,067) used the app after 1 month from the date it was installed, 17.40% (10,108/104,067) used the app after 3 months from the date it was installed, and 5.60% (5828/104,067) used the app after 6 months from the date it was installed.

Click stream data were analyzed to better understand how all users engaged with the app and to evaluate whether usage patterns differed between first-time and returning users (see Table 3). Among those using the app for the first time and who visited a content area (N=73,119), the first content area visited was *mindfulness training* at 63.23% (46,236/73,119), *practice now* at 23.03% (16,837/73,119), *build expertise* at 4.31% (3152/73,119), *track progress* at 2.35% (1719/73,119), or *other* at 6.28% (4594/73,119). Upon initially visiting the app, 29.74% (30,948/104,067) did not visit any of the key content areas of the app, 47.19% (49,110/104,067) visited only a single content area, and 23.07% (24,009/104,067) visited 2 or more content areas. Across all returning visits to the app (N=210,177), the first content areas visited were *mindfulness training* (89,433/210,177, 42.55%), *practice now* (96,026/210,177, 45.69%), *track progress* (10,728/210,177, 5.10%), and *build expertise* (9591/210,177, 4.56%) (see Table 3).

**Table 3:** Detailed session analysis for Mindfulness Coach by first-time users and returning users, from August 2018 to April 2019.

Session analysis		First visit users, n (%)	Returning visit users, n (%)	Between-group differences, *P* value
**First content area visited (first visit users: N=73,119; returning visit users: N=210,177)**				**<.001**
	Badges	581 (0.79)	1430 (0.68)	
	Build expertise	3152 (4.31)	9591 (4.56)	
	Mindfulness training	46,236 (63.23)	89,433 (42.55)	
	Other	4594 (6.28)	2969 (1.41)	
	Practice now	16,837 (23.03)	96,026 (45.69)	
	Track progress	1719 (2.35)	10,728 (5.10)	
**Any content area visited (first visit users: N=107,430; returning visit users: N=272,682)**				**<.001**
	Badges	1481 (1.38)	3399 (1.25)	
	Build expertise	9266 (8.63)	21,709 (7.96)	
	Mindfulness training	52,975 (49.31)	101,636 (37.27)	
	Other	6888 (6.41)	6303 (2.31)	
	Practice now	29,131 (27.12)	115,693 (42.43)	
	Track progress	7689 (7.16)	23,942 (8.78)	
**Number of content areas visited (first visit users: N=104,067; returning visit users: N=340,955)**				**<.001**
	0	30,948 (29.74)	130,779 (38.36)	
	1	49,110 (47.19)	164,507 (48.25)	
	2	16,542 (15.90)	33,084 (9.70)	
	3	5065 (4.87)	8857 (2.60)	
	4	1969 (1.89)	3210 (0.94)	
	5	433 (0.42)	518 (0.15)	

### Change in Mindfulness Mastery Over Time

**Figure 1:**
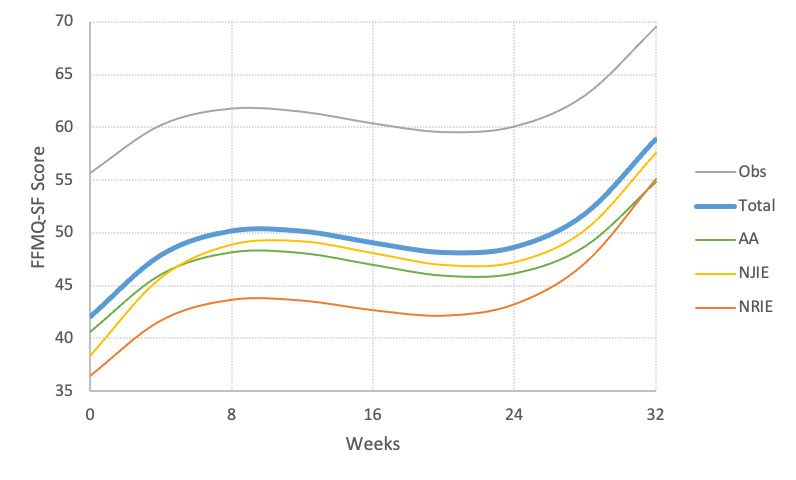
Association between time since installation and being observant (Obs), acting with awareness (AA), nonjudging of inner experience (NJIE), and nonreactivity to inner experience (NRIE). FFMQ-SF: Five-Facet Mindfulness Questionnaire-Short Form.

There was a significant cubic effect of time on FFMQ-SF (*R*^2^=.16, *P*<.001) and each of the 4 subscales: *being observant* (*R*^2^=.06, *P*<.001), *acting with awareness* (*R*^2^=.06, *P*<.001), *nonjudging* (*R*^2^=.08, *P*<.001), and *nonreactivity* (*R*^2^=.07, *P*<.001). Slopes for FFMQ-SF over time rose rapidly between 0 and 8 weeks of app use, leveled off from 8 to 24 weeks, and began to rise again between 25 and 32 weeks after initial use of the app (see Figure 1).

### Mindfulness Mastery as a Function of Engagement With the App

**Figure 2:**
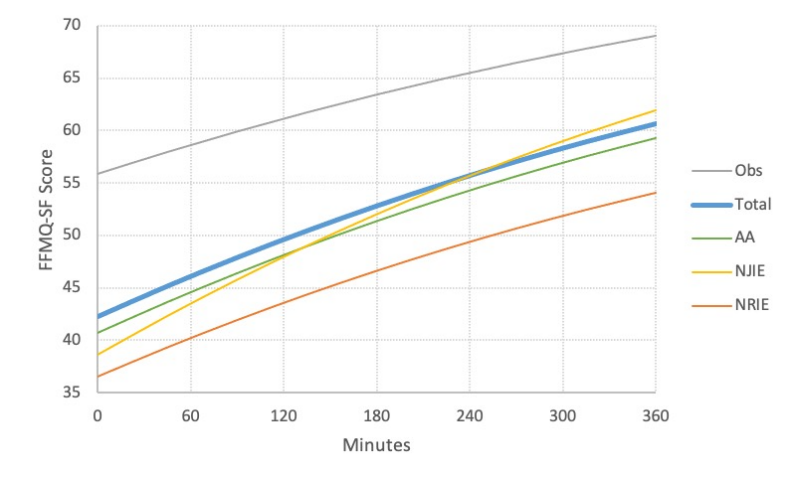
Association between duration of app use and being observant (Obs), acting with awareness (AA), nonjudging of inner experience (NJIE), and nonreactivity to inner experience (NRIE). FFMQ-SF: Five-Facet Mindfulness Questionnaire-Short Form.

There were also strong linear effects of app engagement on total FFMQ-SF scores. FFMQ-SF scores were associated with more hours spent engaged with the app (*R*^2^=.23*, P*<.001) (see Figure 2), total number of visits to the app (*R*^2^=.25, *P*<.001), and number of interactions (ie, events) with the app (*R*^2^=.28, *P*<.001).

## Discussion

### Principal Findings

This natural-use investigation of an mHealth MT app revealed that users vary tremendously in how they use the app in a natural setting. The app was reviewed favorably by users who chose to leave a review, and increased engagement with the app was associated with improved scores on a measure of mindfulness mastery. Because Mindfulness Coach and other similar mHealth mental health apps will be used by many more people over time, this study provides useful information about how the app is used “in the wild.”

Our results revealed the typical use case for mobile app users, which needs to be considered when planning and implementing app-related interventions. Understanding typical use allows mobile interventionists to consider strategies to enhance reach and adherence when creating interventions in this format. While we do not yet know the optimal frequency and duration of use of Mindfulness Coach, this paper helps us understand that many users’ natural inclination is to use the app infrequently and for a short period of time. Mindfulness Coach is currently being examined in open clinical trials, so more information to help understand optimal dose and duration will be forthcoming.

For the highest-engagement group, committed users, the app reached 12,033 users over the span of 8 months. High-engagement users averaged 84 minutes of in-app mindfulness-based training and practice. This suggests that for at least some individuals, the app is highly engaging. Importantly, this study was not able to measure if users were practicing mindfulness and meditating outside of the app. It is possible that mindfulness skills are being transferred and practiced without the app, so our metrics of use are potentially an underestimation of amount of time per week users spend engaged in some type of mindfulness practice (eg, breathing, meditation, additional readings, etc).

Similarly, we found a significant positive relationship between app use and FFMQ-SF scores, suggesting that dedicated users experience improvements in mindfulness characteristics, which may in turn convey improved mental health [2,4]. Other areas of improvement that regular use of the app may be related to needs further research. For example, face-to-face MT has demonstrated benefits for improving pain, depression, anxiety, and quality of life [2]. Does Mindfulness Coach, when used regularly, confer similar benefits? Future research is needed to elucidate the dose response of Mindfulness Coach and the associated benefits of using the mobile app.

Our study indicated that Mindfulness Coach is not being used frequently enough or for a long enough duration by many users. Given that increased Mindfulness Coach use is associated with improved FFMQ-SF scores, it is likely that limited Mindfulness Coach use is not maximally impactful. There was an extreme positive skew in engagement, and nearly 30% of first-time users and 38% of returning users access *only* the home screen, suggesting that users are opening but not using the app. Given that the app is currently used in very low doses by many users, it may be advantageous to couple Mindfulness Coach with face-to-face care, which allows therapy to extend beyond the traditional session. The app, with guidance from a therapist, could be used as a tool to practice and learn mindfulness skills in between face-to-face sessions or, alternatively, as the primary intervention with a therapist checking in less frequently. More research is needed to explore app engagement and corresponding effects as adjuncts to face-to-face therapy. Another use of the app in an intervention could be for someone to check in and prompt people to use the app. Ways to provide structure and to tailor the intervention could help participants receive the full benefits of the app.

Lastly, our study revealed that Mindfulness Coach received excellent ratings in the Google Play Store (Android) and Apple App Store (iOS), signaling that people are very satisfied with the app. Though this is a limited subsample of the user population, it indicates that the intervention is well received by at least some proportion of users. Because an intervention would not be useful or engaging if people were not satisfied with the app, high satisfaction scores are likely necessary but not sufficient in determining whether the app is a beneficial intervention.

### Limitations

While this study provides information on the natural, unguided, and untailored use of the app, we have no information regarding demographics of users. It would be helpful to know who is using the app and if there are populations for whom the app could be more beneficial. Furthermore, we provided a cursory look at the relationship between app use and mindfulness scores as measured by the FFMQ-SF; however, we were unable to explore how app use impacts the well-being, both mental and physical, of the users. Future studies are needed to further explore downstream effects of using Mindfulness Coach, such as quality of life, depression, anxiety, pain, sleep, etc. Additionally, because completing the FFMQ-SF is optional, it is possible that there is bias in the responders who chose to complete the FFMQ-SF. The app does prompt all users to complete the FFMQ-SF assessment, but users are able to close out of the assessment if they choose. Further research will need to validate and confirm this finding in a less potentially biased sample. Another limitation is that assessments were not routinely administered based on time passed, but rather on level achieved or when a user chose to take an assessment. This lack of uniformity in when and if assessments occurred introduces potential for bias. Future studies should look at more uniform assessments at regular time intervals as well as the longitudinal effects of using Mindfulness Coach.

### Conclusions

MT is associated with a broad range of improvements in quality of life and well-being within a range of populations. Mobile apps are accessed regularly by the general population and, thus, represent a potentially ideal way to expand the reach of mental health interventions. The National Center for Posttraumatic Stress Disorder researchers created Mindfulness Coach as a vehicle to deliver mindfulness training, and this study was a first step toward understanding how the app is used, without in-person guidance, by the general population. We looked at data from over 100,000 people and found that the app was used less frequently than the developers and researchers intended by the majority of downloaders, though there was a subpopulation of users (5.60%) who engaged in the app regularly over an extended period of time. We also found a clear relationship between use and improvements in mindfulness as measured by the FFMQ-SF. Future research is needed to understand more specifically who is using the app and how, ways in which we can improve the use of the app, and how to design the app in a way where more users can receive a larger dose of mindfulness treatment.

## Abbreviations

FFMQ: Five-Facet Mindfulness Questionnaire
FFMQ-SF: Five-Facet Mindfulness Questionnaire–Short Form
JSON: JavaScript Object Notation
mHealth: mobile health
MT: mindfulness therapy
VA: Veterans Affairs
